# Identifying Targeted Therapies for CBFA2T3::GLIS2 Acute Myeloid Leukemia

**DOI:** 10.21203/rs.3.rs-6528748/v1

**Published:** 2025-05-13

**Authors:** Fanny Gonzales, Constanze Schneider, Gabriela Alexe, Shan Lin, Delan Khalid, Montserrat Alvarez, Allen Basanthakumar, Jana Ellegast, Lucy Merickel, Silvi Salhotra, Audrey Taillon, Mark Wunderlich, Marc Ansari, Jennifer Perry, Barbara Degar, Yana Pikman, Kimberly Stegmaier

**Affiliations:** Geneva University Medical School; Dana-Farber Cancer Institute; Harvard Medical School; Seattle Children’s Research Institute; Dana-Farber Cancer Institute; Geneva University Medical School; Dana-Farber Cancer Institute; University of Zurich; Dana-Farber Cancer Institute; Dana-Farber Cancer Institute; Dana-Farber Cancer Institute; Cincinnati Children’s Hospital Medical Center; University Hospital of Geneva; Dana-Farber Cancer Institute; Dana-Farber Cancer Institute; Dana-Farber Cancer Institute; Dana-Farber Cancer Institute

## Abstract

*CBFA2T3::GLIS2* fusion positive pediatric acute myeloid leukemia (AML) remains one of the worst prognostic AML subgroups. To uncover innovative targeted therapeutic approaches in this disease subtype we performed genome-scale CRISPR-Cas9 screening that highlighted a strong, selective dependency on *JAK2* compared to other types of cancer.

Using a doxycycline-inducible *JAK2* knockout (KO) system, we validated *JAK2* dependency in *CBFA2T3::GLIS2* cell lines, observing impaired proliferation in vitro and in vivo and induced apoptosis with *JAK2* KO. Both type I (ruxolitinib) and type II (CHZ868) JAK2 inhibitors showed selective in vitro activity in *CBFA2T3::GLIS2* positive AML models.

To identify resistance and sensitizer mechanisms to JAK2 inhibitors, we used CRISPR-Cas9 ruxolitinib anchor screening in *CBFA2T3::GLIS2* AML. sgRNAs targeting negative regulators of the MAPK pathway were enriched in the ruxolitinib-treated cells. Similarly, *CBFA2T3::GLIS2* AML sublines grown to resistance under chronic ruxolitinib treatment expressed pathogenic *NRAS* mutations. Both approaches converged on MAPK pathway activation as a resistance mechanism to ruxolitinib treatment. Combining ruxolitinib with MEK inhibitors showed a synergistic effect in cell lines and patient-derived xenograft (PDX) cells expressing the fusion and in vivo activity in a *CBFA2T3::GLIS2* AML PDX, suggesting a potential approach to target this signaling circuitry in this poor outcome AML subtype.

## INTRODUCTION

Pediatric acute myeloid leukemia (AML) accounts for about 25% of pediatric leukemias and still represents a significant clinical challenge due to its poor prognosis and therapy-related toxicity (1). Acute megakaryoblastic leukemia (AMKL) accounts for ~ 10% of childhood AML and is divided into two subgroups: AMKL arising in patients with Down syndrome, which has an excellent prognosis (~80% survival), and AMKL arising in patients without Down syndrome (non-DS-AMKL), where overall survival is poor (14–34%) despite aggressive chemotherapy and stem cell transplant (2, 3). A recurrent cryptic inversion on chromosome 16, inv (16)(p13.3q24.3), which results in the joining of the genes *CBFA2T3* (CBFA2/RUNX1 partner transcriptional co-repressor 3) and *GLIS2* (GLIS family zinc finger 2), is the most prevalent chromosomal aberration in non-DS-AMKL (~20% patients) (4). Mechanistically, *CBFA2T3::GLIS2* binds DNA through CBFA2T3-associated transcription factors or directly through GLIS2 zinc-finger domains at enhancers and other regulatory elements. *CBFA2T3::GLIS2* binding leads to altered transcription, such as the upregulation of the ETS transcription factor *ERG* and the kinase *KIT*, and the downregulation of genes such as *GATA1*, resulting in a block in differentiation and increased self-renewal capacity (5–7). To date, the exact mechanisms by which the *CBFA2T3::GLIS2* fusion confers leukemia aggressiveness and chemoresistance are not completely understood.

The *CBFA2T3::GLIS2* fusion is an exclusively pediatric AML genomic abnormality, occurring in children under 3 years old, and it is associated with a very poor prognosis, with an overall survival rate of 14% (8). The frequency of co-occurring mutations is low in this subset of leukemia (9, 10), limiting the opportunities for matched targeted therapies. Furthermore, patients with this leukemia are subjected to the same therapeutic protocols as other children with high-risk AML, despite the pronounced chemoresistance observed in this specific disease subset.

One approach to targeting this high-risk subset of AML would be to target the fusion directly. However, targeting fusion transcription factors represents a considerable challenge. To navigate this challenge and investigate new therapeutic opportunities for this aggressive AML subtype, we aimed to find specific dependencies in the context of *CBFA2T3::GLIS2* fusion positive AML by interrogating the Broad Institute’s Cancer Dependency Map (DepMap), a data set composed of genome-scale CRISPR-Cas9 screens in over 1 000 cancer cell lines, and by screening additional *CBFA2T3::GLIS2* positive AML models. We identified the druggable target, *JAK2*, as a strong selective gene dependency and a candidate therapeutic target in *CBFA2T3::GLIS2* AML and identified resistance mechanisms and effective drug combinations with JAK2 inhibitors.

## MATERIALS AND METHODS

### Cell Culture and PDX Samples

Cell lines were obtained from DSMZ (M-07e, MOLM13 and MV4–11) or BioIVT (WSU-AML). The CMS cell line was generously provided by Dr. Soheil Meshinchi (Fred Hutchinson Cancer Center). CMS, MOLM13, MV4–11 and WSU-AML were cultured in RPMI (Mediatech) supplemented with 10% fetal bovine serum (FBS) (Sigma-Aldrich) and 1% penicillin–streptomycin (PS) (Gibco). M-07e was cultured in RPMI supplemented with 10% FBS, 1% PS and 10ng/mL human G-CSF (300 – 23; PeproTech). HEK293T cells were cultured in DMEM (Mediatech) with 10% FBS and 1% PS. During this study, *Mycoplasma* negativity was confirmed using a LookOut Mycoplasma PCR Detection Kit (MP0035; Sigma-Aldrich) and the identity of cell lines was validated through short tandem repeat profiling by the Molecular Diagnostics Laboratory at the Dana-Farber Cancer Institute (DFCI).

Primary patient samples were previously acquired following written informed consent in accordance with the Declaration of Helsinki, and patient-derived xenografts (PDXs) were established under protocols approved by the Dana-Farber Cancer Institute (DFCI) and Cincinnati Children’s Hospital Medical Center (CCHMC) Institutional Review Boards (11–13). For short-term in vitro culture, PDX cells were maintained in Iscove’s modified Dulbecco’s medium (Mediatech) containing 20% FBS and 1% PS and supplemented with 10ng/mL human SCF, TPO, FLT3L, IL3, and IL6 (300–07, 300 – 18, 300 – 19, 200–03, and 200–06; PeproTech). Detailed information on AML cell lines and PDXs used for this study are provided in Supplementary Table S1.

AML and PDX cells were transduced with lentivirus co-expressing Cas9 and a fluorescent protein (mCherry) (Addgene plasmid #70182). Cas9-expressing cells were selected based on mCherry fluorescence. Cas9-expressing PDX cells were expanded via serial transplantation into immunodeficient NSGS (NOD scid gamma SGM3) mice. Cas9 activity was assessed using an EGFP reporter pXPR_047 (107145; Addgene).

### CRISPR-Cas9 Screens

AML cells stably expressing *Streptococcus pyogenes* Cas9 (CMS and WSU-AML) were transduced in duplicate with the Broad Institute’s AVANA-4 sgRNA library (20,21) which contains 74,687 unique sgRNAs with ~4 sgRNAs per gene at an MOI of ~30%. Puromycin selection was initiated on the day after transduction and maintained for 3 days until selection was complete. For the drop-out screens, cells were expanded for 21 days in culture and passaged every 4 days. For the drug modifier screens, after selection, cells were divided into two treatment arms and treated for 14 days with DMSO or ruxolitinib (150 nM for CMS and 50 nM for WSU-AML), with two replicates each. Representation of 500–1000 cells per guide was achieved and maintained throughout the screen. Genomic DNA was extracted from the pellets using the NucleoSpin Blood L kit (Takara #740954.20) 21 days post infection. The sgRNA barcodes were PCR amplified, and the product submitted for standard Illumina sequencing as previously described (16).

### Dependency Data Analysis for the Drop-out and Drug Modifier CRISPR-Cas9 Screens

The WSU-AML drop-out screens and the CMS and WSU-AML CRISPR-Cas9 drug modifier screens were sequenced on the Genetic Perturbation Platform (GPP) at the Broad Institute (https://www.broadinstitute.org/genetic-perturbation-platform). The sgRNAs read count data were deconvoluted from sequencing reads by using the PoolQ software (https://portals.broadinstitute.org/gpp/public/software/poolq). The AVANA-4 library annotation files for sgRNAs efficacy (CRISPRInferredGuideEfficacy.csv) and common essential genes (CRISPRInferredCommonEssentials.csv, AchillesCommonEssentialControls.csv) were downloaded from the 24Q4 DepMap public database https://depmap.org/portal/download/ (17). The sgRNA reads were log-normalized to the initial plasmid pool (pDNA).

Quality control pre-processing steps were performed to filter out the guides participating in the fusion by removing the fusion guided GLIS2 ATACTCAGCTTCAGGTCGAG, GLIS2 GAGTATCACCAAGCTCCGGG and keeping the non-fusion guides GLIS2 TCAAGCCCGAGAAGGATGCG and GLIS2 ACACCATCCAAATAGCGCAG, and by removing the guides that have low efficacy (≤ 0.35) and the guides with low representation in the initial plasmid pool (pDNA). The sgRNA guides that passed the filtering were mapped to genes based on the CP0033_GRCh38_NCBI_CRISPRko_strict_gene_20221209.chip annotations. Exogenously defined nonessential genes for the AVANA-4 library were used as negative controls, and common essential genes were used as positive controls in further quality control steps. All samples passed the QC tests, except for the Replicate 2 CMS ruxolitinib (150 nM)-treated sample, which was disregarded.

The gene effect dependency scores for the WSU-AML drop-out CRISPR screens and for the CMS and WSU-AML DMSO-treated day 14 vs. day 0, CMS and WSU-AML ruxolitinib-treated day 14 vs. day 0 and the differential dependency scores for CMS and WSU-AML ruxolitinib vs. DMSO treated cells at day 14 were computed from the sgRNA log-norm data based on the hypergeometric distribution tool (https://portals.broadinstitute.org/gpp/public/). The magnitude of the gene-level dependency effect was inferred as the average log-norm dependencies of the sgRNAs assigned to the gene. The p-values were computed using the probability mass function of a hypergeometric distribution for the log-norm dependency-ranked sgRNAs. Significance was estimated based on the cut-offs abs (average log-fold change) ≥ 1.5 and p-value ≤ 0.05. Genes with negative differential dependency scores in ruxolitinib vs DMSO treated cells were classified as “drug sensitizers,” meaning their knock-out (KO) is associated with increased drug sensitivity. Conversely, genes with positive differential dependency scores were associated with drug resistance, indicating that their KO is associated with decreased drug sensitivity.

The CRISPR dependency data for the fusion-positive AML cell lines CMS and M-07e was obtained from the public 24Q4 DepMap database (https://depmap.org, CRISPRGeneEffect.csv and AvanaLogfoldChange.csv files). Gene effect dependency was estimated based on the Chronos gene effect score for all genes, except *GLIS2*. For *GLIS2* the gene effect was estimated as the average Avana log fold change for the two *GLIS2* guides not participating in the fusion TCAAGCCCGAGAAGGATGCG and ACACCATCCAAATAGCGCAG. Gene effect scores ≤ −0.5 were evaluated as significant dependencies.

### Lentivirus Production and Transduction

For the CRISPR guides targeting *JAK2*, two guide sequences were retained from the AVANA-4 library (sg*JAK2*-1, ATCTGCCTCAGATTTCCCAA; sg*JAK2*-2, GAGGTGCTACTTCTTTACAG) and one sgNT (sgNT, CCGCGCATTTCAGAGCACAA) was used as a non-targeting control. For this study, we used inducible CRISPR, by cloning sgRNAs of interest in a guide only vector containing a doxycycline inducible sgRNA and constitutive GFP, FgH1tUTG, obtained from Addgene (# 70183; http://n2t.net/addgene:70183; RRID:Addgene_70183), as previously described (18). The Cas9-T2A-mCherry vector was also obtained from Addgene (#70182). For *RASA2* KO, sg*RASA2*-1 (TTAGCATCAAGGCATGCCAT) and sg*RASA2*-2 (GCCTGTTGACTCCAATTCAG) were cloned into pXPR_003 (19).

Lentiviral particles were generated by co-transfecting cells with lentiviral expression vectors along with viral packaging plasmids pCMV-VSV-G (Addgene #8454) and psPAX2 (Addgene #12260) into HEK293T cells using Fugene transfection reagent (Promega). For transduction, AML cells were mixed with viral supernatant and 8μg/mL polybrene. Cells were centrifuged in viral supernatant at 2,000 × rpm for 2 hours at 25°C to enhance the transduction efficiency. All experiments were performed with polyclonal cell populations.

### Confirmation of sgRNA Editing

Gene KO was confirmed by immunoblotting for the respective targets (JAK2/pSTAT3/pSTAT5) for *JAK2* KO or *RASA2* experiments. Evaluation of gene KO efficiency was performed 4 days after induction by doxycycline.

For in vivo experiments, on-target editing of *JAK2* was also confirmed by amplifying the appropriate region of genomic DNA and then performing Sanger sequencing with deconvolution by the TIDE (tracking of indels by decomposition) or ICE (inference of CRISPR edits) algorithms. For *JAK2*, the region targeted by all guides was amplified using the following primers: Forward 5’ ACTGAGCCATAAAAGATATGAGCAA 3’ Reverse 5’ ACTGTGGCTTCCTTGCAGTA 3’. PCR products were purified using the QiaQuick PCR purification kit and sent for Sanger sequencing with the following sequencing primers: sg*JAK2* 5’ ACTGAGCCATAAAAGATATGAGCAA 3’. On-target editing was then determined using either the TIDE algorithm https://tide.nki.nl/ (20) or ICE v. 2.0 by Synthego (https://ice.synthego.com/#/) as indicated.

### Competition Assays

Cells were transduced with lentiviral vectors co-expressing an sgRNA and the green fluorescent protein (GFP) at an efficiency of approximately 50%, or the transduced cells were mixed with non-transduced cells at approximately a 1:1 ratio. The cell growth was evaluated by the change in the fraction of cells expressing GFP, which was monitored by flow cytometry from the day of induction to 3 weeks post D0. D0 was defined as 4 days post induction by doxycycline.

### Cell Viability

To determine the effects of *JAK2* deletion on cell viability in *CBFA2T3::GLIS2* fusion AML, cells were sorted for the GFP + population, expanded and plated in 384-well plates (Corning #3570) at a concentration of 500 cells per well in 50 μL of cell culture medium. Cells were assessed for viability on day 0 and subsequent time points (D3, 5 and 7) using the CellTiter-Glo luminescent assay kit (Promega) according to the manufacturer’s protocol (10 μL/well). Luminescence was read on a Fluostar Omega Reader (BMG Labtech), and the viability was normalized to day 0.

### Flow Cytometry

Apoptosis was analyzed with an APC Annexin V Apoptosis Detection Kit (BioLegend) following the manufacturer’s protocol. Cell cycle status was analyzed using a Click-iT Plus EdU Alexa Fluor 647 Flow Cytometry Assay Kit (Invitrogen #C10634) following the manufacturer’s protocol. Cells were incubated with 10 μM EdU for 1.5 hours, and the DNA content was co-stained with FxCycle Violet (Invitrogen #F10347).

Mouse samples were stained with V450 anti-human CD45 and APC-Cy7 anti-mouse CD45 (BD Biosciences #560368 and #557659) antibodies. Cells were analyzed on an LSR Fortessa or FACS Celesta flow cytometer or sorted on a FACS Aria II flow cytometer (BD Biosciences), and the data were analyzed with FlowJo software (TreeStar).

### Xenograft Transplantation

All animal experiments were approved by the DFCI Institutional Animal Care and Use Committee with adherence to all appropriate guidelines. Transplantation was performed on 6- to 8-week-old NOD/SCID/IL2RG^−/−^ immunodeficient mice with transgenic expression of human SCF, GM-CSF, and IL3 (NSGS; The Jackson Laboratory).

For doxycycline-inducible sg*JAK2* experiments, 1 × 10^6^ Cas9-expressing WSU-AML cells transduced either with sgNT or *sgJAK2*-2 and GFP-sorted were transplanted into each mouse via tail vein. Doxycycline-containing food (625mg/kg) was initiated after engraftment was confirmed by bone marrow aspirations (BMA). For the experiment evaluating ruxolitinib treatment, mice were treated with 0.5% methylcellulose solution or ruxolitinib (60mg/kg/dose) twice daily (5 days per week) by oral gavage for 2 weeks as previously reported (21). Ruxolitinib was purchased from LC Labs (#R-6600) for in vivo use and selumetinib from Shanghai Medicilon. For the four-arm study, mice were randomly assigned to receive vehicle, ruxolitinib (60mg/kg/dose) twice daily (5 days per week), selumetinib reconstituted in 0.5% HPMC + 0.1% Tween 80 (50mg/kg/dose) twice daily (5 days per week) or the combination of ruxolitinib and selumetinib.

For both experiments, leukemia burden was assessed by determining the chimerism of the human isoform of the pan-leukocyte marker CD45 in peripheral blood, bone marrow, spleen and measurements of hematopoietic organs (spleen/liver) 2 weeks after treatment.

### Western Blotting

Cells were lysed in RIPA Lysis and Extraction Buffer (Thermofisher #89900) supplemented with Halt^™^ Protease Inhibitor Cocktail (Thermofisher #78429) and phosphatase inhibitors (Roche #04906845001). Lysates were quantified using a Bradford protein assay (Bio-Rad) and normalized. SDS-PAGE gels were used to separate proteins, and proteins were transferred to a PVDF membrane. The following primary antibodies were used from Cell Signaling Technology (CST): JAK2 (D2E12) (#3230S), pJAK2 (Tyr1007/1008) (#3771S), pSTAT3 (Tyr705) (#9145S), STAT3 (124H6) (#9139), pSTAT5 (Tyr694) (#9351), STAT5 (D2O6Y) (#94305S), beta-Actin (#4967L), MEK1/2 (L38C12) (#4694S), pMEK1/2 (Ser217/221) (#9154), AKT (#9272S), and pAKT (Ser473) (#4060), pERK (Thr980) (#16F8), p44/42 MAPK (Erk1/2) (127F5) (#4695) and vinculin (#4650). The primary antibody against RASA2 was purchased from Sigma Aldrich (HPA035374). Membranes were incubated with secondary antibodies (HRP-linked goat anti-rabbit IgG and anti-mouse IgG (CST #7074, #7076) and imaged on an Amersham^™^ Imager 680 (GE Healthcare).

### Drug Response Testing

AML and PDX cells were seeded in 50 μl per well in 384-well plates (Corning #3570) at the density of 100,000cells/ml for AML cells and 200,000 cells /ml for PDX cells. Compounds were added alone or in combination to the wells, with four replicates for each dose or dose pairing with a HP D300 Digital Dispenser. DMSO was normalized to the highest DMSO volume added for the entire plate, not to exceed 0.02%/well. Cells were analyzed for cell viability after 3 (JAK inhibitors) to 5 days (MEK inhibitors) of treatment using the CellTiter-Glo luminescent assay (Promega #G7571) following the manufacturer’s instructions. Ruxolitinib (#S1378), tofacitinib (#S2789), trametinib (#S2673) and selumetinib (#S1008) were purchased from Selleck Chemicals for in vitro use. CHZ868 was purchased from MEDCHEM Express LLC (#HY-18960). The drug response curves for baseline-corrected, normalized log(concentration) data were fitted based on the non-linear regression equation log(inhibitor) vs. response ~ variable slope (four parameters). The Extra-sum-of-squares F-test was used to test the hypotheses if one curve adequately fits all the datasets and if the best fit of LogIC50 is the same among the datasets. The difference in the overall efficacy of the drug across the complete range of concentrations in the fusion vs. the non-fusion cell line groups was estimated based on the two-tailed t-test applied to the Area under Curve scores for the fitted curves in the two groups.

### Synergy Analysis

To determine if individual treatment combinations were synergistic, additive, or antagonistic, we calculated the Combination Index (CI) scores using the Chou-Talalay Median Effect model (22–24). The CI score quantifies the effect *x* produced by the combination of dose d 1 of drug 1 and dose d 2 of drug 2: CI=d1/D*x*1+d2/D*x*2, where D*x*1 is the dose of drug 1 that alone produces the effect *x* and D*x*2 is the dose of drug 2 that alone produces the effect *x*, as estimated from the median effect model. For any endpoint of the effect measurement, Cl estimates the following interactions:

- Strong synergism: log 10(CI) ≤ −0.22
- Synergism: log 10(CI) > −0.22 and ≤ −0.10
- Additivity: log 10(CI) > −0.10 and < 0.08
- Antagonism: log 10(Cl) ≥ 0.08 and < 0.20
- Strong antagonism: log 10(Cl) ≥ 0.20

The fractional inhibition effect for drug combinations was visualized using heatmaps, and the combination index scores versus the fractional inhibition effect were presented as scatter dot plots.

### Generation of Resistant Cell Lines

To generate cells that were resistant to JAK inhibition, parental CMS and M-07e cells were treated for 6 months with gradually increasing concentrations of ruxolitinib (10 nM to 1.5 μM). Cells were considered ruxolitinib-resistant when they were able to remain 90–100% viable in the presence of ruxolitinib at a concentration 10-fold higher than the IC_50_ of the parental cells.

### Statistics

The statistical tests used, and *P*-values calculated, are described in the figure legends. All t-tests are unpaired and two-sided unless otherwise indicated. A *P*-value of 0.05 was used as the cutoff for significance unless otherwise indicated. Statistical tests were calculated in GraphPad Prism 10.0.2.232. Error bars represent SD unless otherwise indicated. All duplicate measures were taken from distinct samples rather than repeated measures of the same sample.

## RESULTS

### CRISPR-Cas9 screens in three *CBFA2T3::GLIS2* cell lines revealed JAK2 as a strong selective dependency

**Figure 1:**
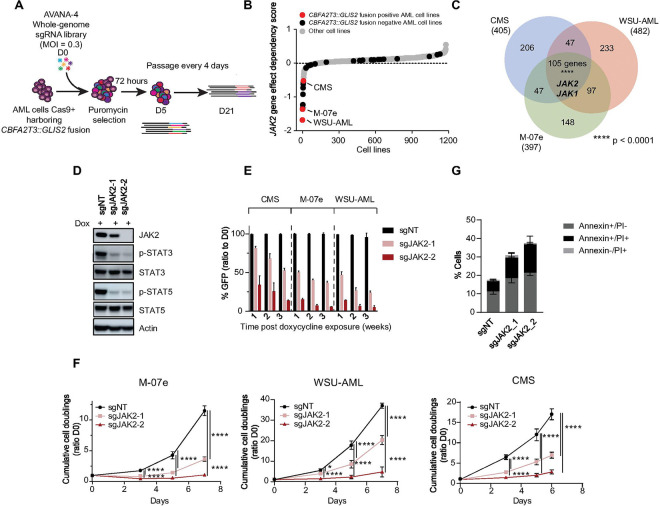
Legend not included with this version

Genome-wide CRISPR-Cas9 KO screening has been deployed to identify critical gene dependencies in cancer (25, 26). Using this approach (Fig. 1A), more than 1 000 cancer cell lines have been screened in the context of the Cancer Dependency Map (17, 27) (https://depmap.org/portal), including two *CBFA2T3::GLIS2* fusion AML cell lines (CMS and M-07e) and 26 *CBFA2T3::GLIS2* non fusion AML cell lines (12). Expanding upon these data, we performed genome-scale screens in an additional cell line harboring the fusion, WSU-AML (derived from the same patient’s AML as M-07e). All together, these screens revealed 105 genes as top dependencies commonly shared by the three cell lines harboring the *CBFA2T3::GLIS2* fusion, including *JAK2* and *JAK1* (Figs. 1B, C, Supplemental Figure S1A, Supplemental Table S2).

### JAK2 depletion impaired cell viability and induced apoptosis in vitro

Because JAK2 was a stronger hit in 2 of the 3 screens than JAK1, we focused our attention on JAK2 (Supplemental Figure S1A). We used a doxycycline-inducible KO (Dox-KO) system and two distinct sgRNAs targeting *JAK2*. We first validated that KO of *JAK2* led to a decrease in JAK2 protein levels and impaired the downstream signaling of the kinase with lower levels of phosphorylated STAT3 and STAT5 observed by western blotting (Fig. 1D). Next, we validated the dependency on *JAK2* in the three AML cells lines harboring the *CBFA2T3::GLIS2* fusion. *JAK2* KO suppressed the growth of AML cells, measured by an in vitro competition assay (Fig. 1E) and by cumulative cell doubling measurements (Fig. 1F), with a significant increase in Annexin V positive cell death (Fig. 1G). *JAK2* KO also impaired cell cycle distribution with a reduced S phase and increased G0/G1 phase (Supplemental Figure S1B). There was no difference in differentiation as measured by CD11b or CD41 staining in cells with *JAK2* KO (Supplemental Figure S1C).

### JAK2 is essential for AML progression in vivo

**Figure 2:**
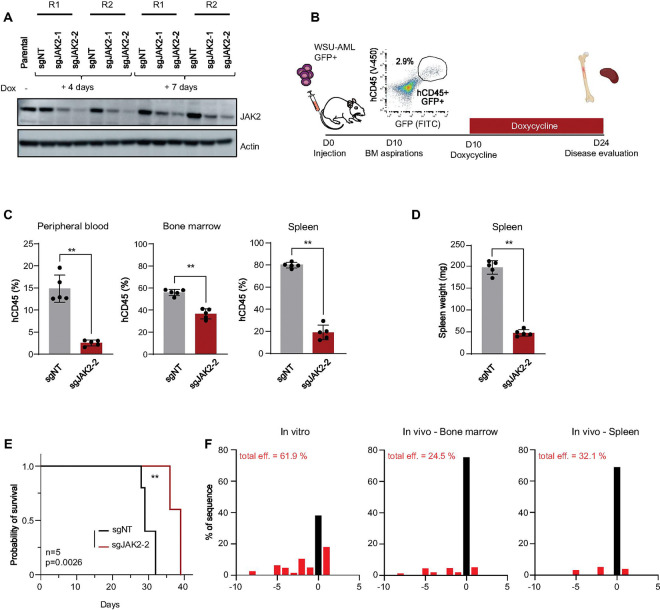
Legend not included with this version

We next asked whether disruption of *JAK2* can impair AML progression in vivo. WSU-AML cells were transduced with the Dox-KO (non-targeting control (sgNT) or *JAK2*-targeting sgRNAs) vectors co-expressing GFP. Before injection, KO efficiency was confirmed by immunoblotting for JAK2 after doxycycline induction in vitro (Fig. 2A). We chose the CRISPR guide with the greatest KO efficiency to evaluate in vivo. FACS-sorted GFP + sgNT and sg*JAK2*-2 cells were intravenously injected into non-irradiated NSGS mice. BMA performed on day 10 showed significant engraftment with an average of 1.2% human CD45/GFP positive cells (n=4 samples). A doxycycline diet was initiated on day 10 to induce KO (Fig. 2B). After 2 weeks of doxycycline chow, mice with *JAK2* KO cells had a significantly reduced leukemia burden in bone marrow, spleen and peripheral blood (Fig. 2C), as well as decreased spleen weight (Fig. 2D). *JAK2* KO led to prolonged survival compared to controls (39 versus 29 days, p = 0.0026) (Fig. 2E). At sacrifice, disease burden evaluation based on percentage of human CD45 cells did not show any difference between the two groups, suggesting that the cause of death was ultimately from progressive leukemia (Supplemental Fig. 2A). TIDE and ICE analyses performed on bone marrow and spleen samples collected at study endpoint showed that only a minor population of these cells retained *JAK2* locus editing (Fig. 2F). Immunoblotting of leukemia cells in the bone marrow and spleen did not show a decrease in JAK2 protein levels (Supplemental Fig. 2B) consistent with a strong selection pressure against the deletion of *JAK2* in vivo. Therefore, AML cells driving progressive disease had escaped *JAK2* deletion, consistent with an essential role for JAK2 in *CBFA2T3::GLIS2* AML progression in vivo.

### Primary patient *CBFA2T3::GLIS2* AML is enriched for JAK-STAT transcriptional programs

**Figure 3:**
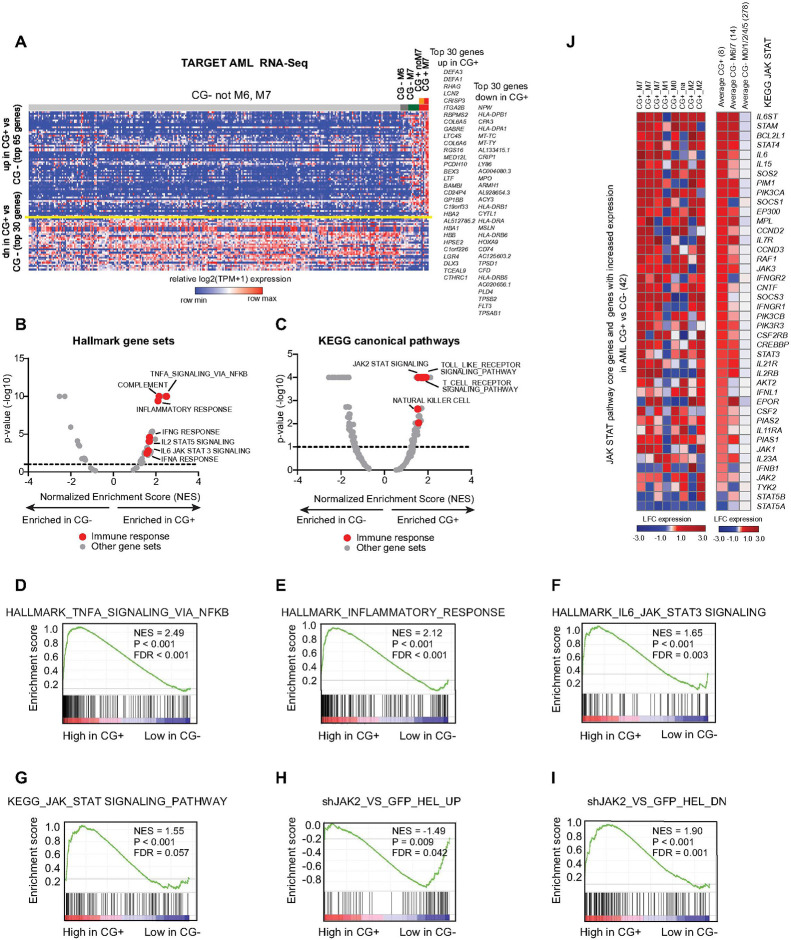
Legend not included with this version

We next examined RNA sequencing data from the TARGET AML cohort including 8 *CBFA2T3::GLIS2* fusion positive samples compared to 292 AML samples without the fusion (Fig. 3A, Supplemental Table S3). Gene set enrichment analysis (GSEA) showed upregulation of several inflammatory signaling pathways including TNF-α signaling via NF-⊠B, IL2-STAT5, IL6-JAK-STAT3 and JAK2-STAT signaling in the *CBFA2T3::GLIS2* fusion positive samples (Figs. 3B–G). Furthermore, the fusion positive samples were enriched in a JAK2 shRNA knockdown signature published by Rampal et al. (GEO GSE54645), that was obtained from *JAK2*V617F homozygous mutant HEL cell line following treatment with two independent shRNAs targeting JAK2 (28) (Fig. 3H–I). We next sought to explore whether the enrichment of the JAK-STAT gene set was driven by the fusion, cell lineage, or both. To this end, we compared the expression of the JAK-STAT signature genes in primary *CBFA2T3::GLIS2* positive AML (n=8), versus M6-M7-AML-non-*CBFA2T3::GLIS2* AML (CG- M6, CG- M7, n = 14), versus non-M6-M7-non-*CBFA2T3::GLIS2* AML (CG- not M6, not M7, n=278). We observed that the JAK-STAT related genes were the most highly expressed in the *CBFA2T3::GLIS2* positive AML. However, a subset of them were also upregulated in M6 and M7-AML that is *CBFA2T3::GLIS2* fusion negative (Fig. 3J), suggesting that there is both a fusion-related and a lineage-related contribution to the hyperactivation of these genes. To further evaluate this observation, we analyzed the enrichment scores for the KEGG JAK-STAT pathway in *CBFA2T3::GLIS2* fusion-positive samples, either M7 or non-M7, compared to *CBFA2T3::GLIS2* fusion-negative samples. We found a significant enrichment for fusion-positive M7 patients and a borderline significant enrichment for fusion-positive non-M7 patients (Supplemental Figs. 3A-B). However, the same pathway was not significantly enriched in *CBFA2T3::GLIS2* fusion-negative M6/M7 AML samples versus fusion-negative non-M6/M7 AML samples (n = 14) (Supplemental Fig. 3C). More broadly, by performing single sample GSEA (ssGSEA) on the MSigDB hallmark gene sets, we found that the *CBFA2T3::GLIS2* fusion positive M7 and non-M7 AML samples and to a lesser extent the *CBFA2T3::GLIS2* fusion negative M7 samples shared an enrichment in inflammatory gene signatures, including “Inflammatory response” and “IL6_JAK_STAT3_signaling” (Supplemental Fig. 3D). This pattern was also observed in a similar ssGSEA performed on the KEGG pathways and Gene Ontology Molecular Function gene sets (Supplemental Figs. 3E). In all cases, the *CBFA2T3::GLIS2* M7 AML samples showed the strongest enrichment.

Based on these observations, we hypothesized that *CBFA2T3::GLIS2* AML cells would be sensitive to therapeutic interventions targeting the JAK-STAT pathway.

### *CBFA2T3::GLIS2* AML responds to JAK2 small molecule inhibitors

The type I JAK1/2 inhibitor ruxolitinib is approved by the US Food and Drug Administration (FDA) for treatment of myeloproliferative neoplasms (29,30) and steroid-refractory acute graft-versus-host disease (GvHD) in patients 12 years and older (31). Moreover, type II JAK2 inhibitors, such as CHZ868, have been developed. These type II inhibitors bind and stabilize JAK2 in the inactive conformation and do not lead to an accumulation of phosphorylated JAK2 as seen with type I inhibitors (32, 33). Because of the clinical translatability of ruxolitinib, we largely focused on this inhibitor in our subsequent studies in models of *CBFA2T3::GLIS2* AML.

**Figure 4:**
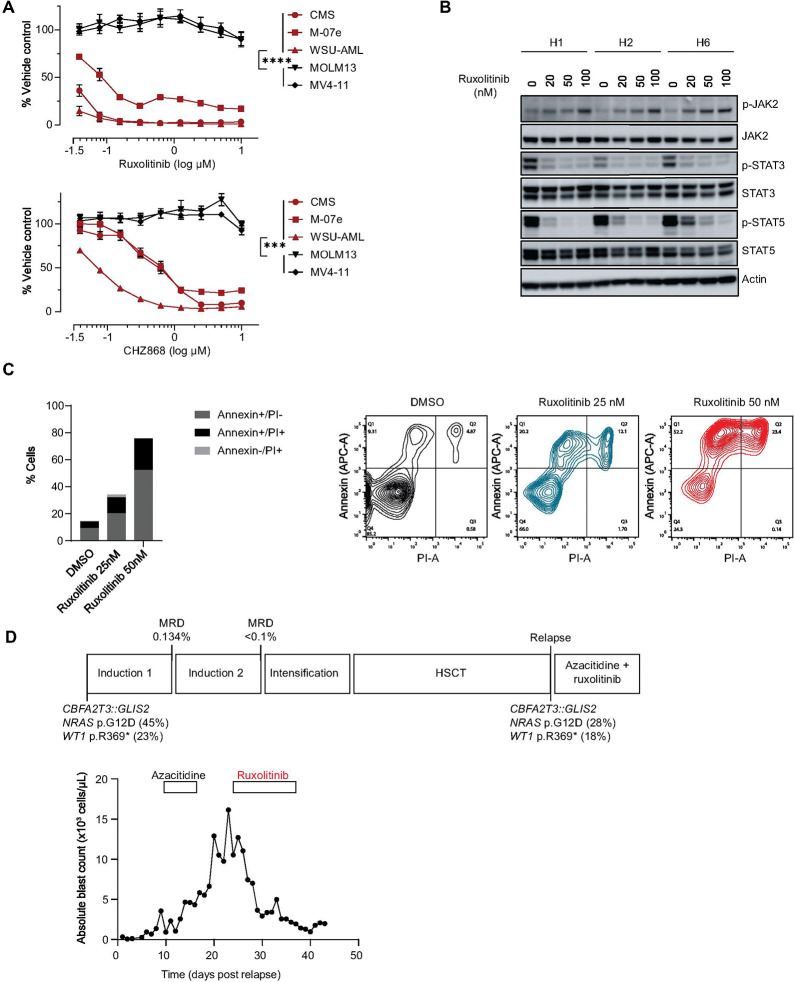
Legend not included with this version

Three cell lines with the *CBFA2T3::GLIS2* fusion were responsive to ruxolitinib, CHZ868 and tofacitinib (type I inhibitor) in contrast to other AML models lacking the fusion, such as MOLM13 or MV4–11 (Fig. 4A and Supplemental Fig. 4A). As expected, ruxolitinib treatment decreased pSTAT3 and pSTAT5 while increasing JAK2 phosphorylation levels, as previously reported (34, 35) (Fig. 4B). Ruxolitinib treatment also led to Annexin V positive cell death (Fig. 4C).

We next treated a pediatric patient with relapsed, refractory *CBFA2T3::GLIS2* AML post allogeneic stem cell transplantation (HSCT) with ruxolitinib. The goal of this intervention was to provide both potential anti-AML activity and GvHD prophylaxis. The patient was initially diagnosed with AMKL at 14 months of age when he presented with fever, pallor and fatigue. His peripheral blood white blood cell count was 75 K cells/μL with 66% blasts. Flow cytometric analysis of his bone marrow was diagnostic for AML with a RAM phenotype (36). Molecular analyses showed the presence of a *CBFA2T3::GLIS2* fusion as well as an NRAS p.G12D with a variant allele frequency (VAF) of 44.9% and a *WT1* p.R369* (VAF 23%). He was enrolled on the AML16 clinical trial: a Phase 2 Trial of Epigenetic Priming in Newly Diagnosed AML (NCT03164057) and was randomized to arm B (decitabine). He achieved remission with positive MRD (0.134% by flow cytometry) after Induction I chemotherapy and MRD below 0.1% threshold after Induction II chemotherapy. Based on adverse biologic features, he was assigned to the high-risk group and proceeded to HSCT after 3 cycles of multiagent chemotherapy. He underwent an umbilical cord blood transplant (HLA matched 5/6) after a myeloablative conditioning regimen consisting of busulfan, cyclophosphamide and Anti-Thymocyte Globulin. The post-transplant period was marked by the occurrence of GvHD.

At 89 days post transplantation, myeloid blasts appeared in the peripheral blood and a bone marrow analysis showed recurrent AML. Molecular abnormalities present at initial diagnosis were maintained with a *CBFA2T3::GLIS2* fusion, NRAS p.G12D (VAF 28%) and *WT1* p. R369* (VAF 18%). He initiated azacitidine with palliative intent and experienced rising disease burden during the first week of this therapy. Ruxolitinib was added for both AML treatment and GvHD prophylaxis. With the introduction of ruxolitinib, the absolute blast count in the peripheral blood decreased and stabilized (Fig. 4D). Unfortunately, his AML ultimately progressed, and the patient died of refractory AML 80 days after his relapse. This clinical case highlights the need to understand resistance mechanisms and develop drug combinations to improve efficacy in this very aggressive AML subtype.

From this patient’s AML cells obtained from the first relapse, we developed a PDX model through serial transplantations in NSGS mice (CPCT-0027)(11). Mutational analysis confirmed that the PDX retained the same genetic alterations as the primary patient sample. We utilized this model to test JAK inhibition using ruxolitinib and tofacitinib, comparing cells briefly cultured in vitro to that of cells from three other PDX models: one harboring the *CBFA2T3::GLIS2* fusion (2016–35) and two lacking this fusion (16 – 01 and 17 – 14) (AML model characteristics in Supplemental Table S1). For both drugs, we observed a better response in the PDX cells harboring *CBFA2T3::GLIS2* fusion compared to the PDX lacking the fusion (Supplementary Fig. 4B).

### Genome-scale CRISPR-Cas9 screen identified activation of MAPK pathway as a resistance mechanism to JAK2 inhibition

To identify candidate resistance mechanisms and potential synergistic combinations with JAK2 inhibitors, we utilized CRISPR-Cas9 screening in combination with ruxolitinib in CMS and WSU-AML. We chose two fusion positive cell lines with different collaborating genetic events (Supplemental Table S1) and likely different myeloid lineage (CMS is more monocytic with higher CD11b expression, and WSU-AML is more megakaryocytic with higher CD41/CD42 expression) (Supplemental Fig. 5A).

**Figure 5:**
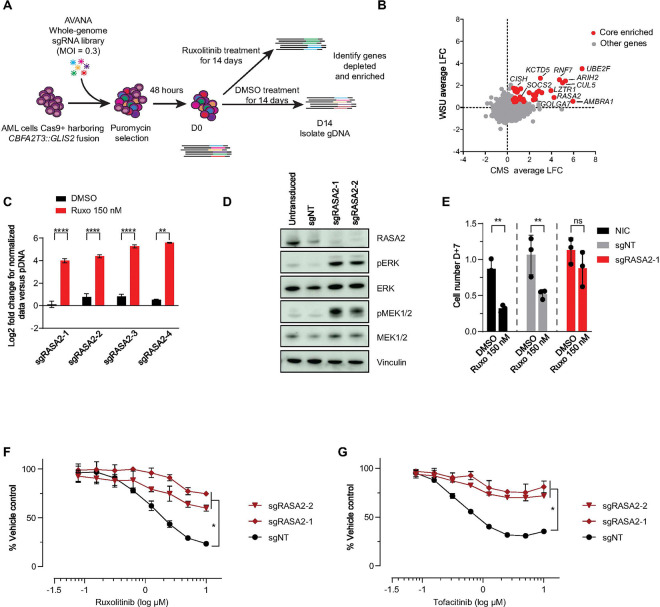
Legend not included with this version

For each cell line, we confirmed that Cas9 expression did not substantially alter the response to ruxolitinib (Supplemental Fig. 5B and C). Then, we determined a concentration of ruxolitinib which reduced cell viability to approximately 50% but still maintained effective inhibition of JAK2, as evidenced by a decrease in STAT3 and STAT5 phosphorylation (Supplemental Fig. 5D). We then transduced cells expressing constitutive Cas9 with the AVANA-4 library as previously described (14). Cells were selected using puromycin and treated with either DMSO or ruxolitinib. Cell viability upon treatment with ruxolitinib was assessed at each passage and corresponded to the expected dose-response pre-established for each cell line (Supplemental Fig. 5E). We next performed sequencing of the sgRNAs to assess the relative abundance of sgRNAs in ruxolitinib-treated and DMSO-treated cells after 14 days of treatment (Fig. 5A).

We were interested in discovering genes that modify response to JAK2 inhibition and selectively alter growth in ruxolitinib treated cells compared to DMSO treated cells. For each cell line, we calculated the log2 fold change of the abundance of each sgRNA in the ruxolitinib- and DMSO-treated conditions relative to the plasmid pool. We then assessed the differential sensitivity in log2 fold change for each sgRNA in the ruxolitinib- compared to the DMSO-treated cells and calculated the average for each gene. There was a strong correlation in differential log2 fold change between the two cell lines screened, which provided confidence in the robustness of the screening results (Fig. 5B). We then used the hypergeometric test to identify genes that had significantly different effects in the JAK2 inhibitor arm compared to the DMSO arm. Our screen was designed to identify genes whose depletion made the cells more resistant or more sensitive to ruxolitinib treatment. However, genes whose depletion rendered resistance scored much more strongly than did sensitizers. Thus, we focused on the genes whose loss promotes resistance. Among the top genes on the resistance side were negative regulators of the JAK-STAT pathway: the Suppressors of Cytokine Signaling (SOCSs) family members *CISH* and *SOCS2*, as well as Cullin 5 (*CUL5*). The SOCS proteins interact with the elongation protein B/C complex through its C-terminal SOCS box and simultaneously bind to the Cullin 5 scaffold protein to form an elongation protein-cullin-SOCS3 E3 ubiquitin-linked enzyme complex. This complex undergoes polyubiquitination, and the proteasome degrades signaling factors such as JAKs and STATs that bind to SOCS, thereby blocking signal transduction (37, 38). Loss of genes belonging to the SOCS family occurring either through germline mutations leading to haploinsufficiency (*SOCS1*) (39) or through the use of shRNA (40) are associated with JAK-STAT pathway activation. These results provide further confidence that the screen data was robust.

Among the top scoring novel hits were multiple genes involved in the negative regulation of the RAS-MAPK pathway, such as *RASA2*, *GOLGA7* and *LZTR1*. We focused on *RASA2* (RAS p21 protein activator 2), a gene recurrently mutated by loss-of-function mutations in the RASopathy Noonan syndrome (41), and in a number of other malignancies (42, 43), as all 4 guides scored in our screen for the CMS cell line (Fig. 5C) and one for WSU-AML (Supplemental Figure S5F). We knocked-out *RASA2* in the CMS cell line, a *CBFA2T3::GLIS2* fusion cell line without known mutations in the RAS-MAPK pathway. *RASA2* KO increased pERK and pMEK expression as a surrogate of MAPK pathway activation (Fig. 5D). KO of *RASA2* alone did not affect cell growth compared to the control (sgNT) or non-infected cells (NIC) in short-term experiments. The *RASA2* KO cells, however, were more resistant to ruxolitinib (Figs. 5E and F). In further validation of these findings, *RASA2* KO also rendered CMS cells resistant to type I JAK inhibitor tofacitinib (Fig. 5G). Together, these data support the notion that the activation of the MAPK pathway, including through the loss of negative regulators of RAS (e.g., *RASA2*) can render resistance to JAK inhibitors.

### Generation of resistant cell lines revealed acquired NRAS mutations as a mechanism of resistance to JAK inhibition

**Figure 6:**
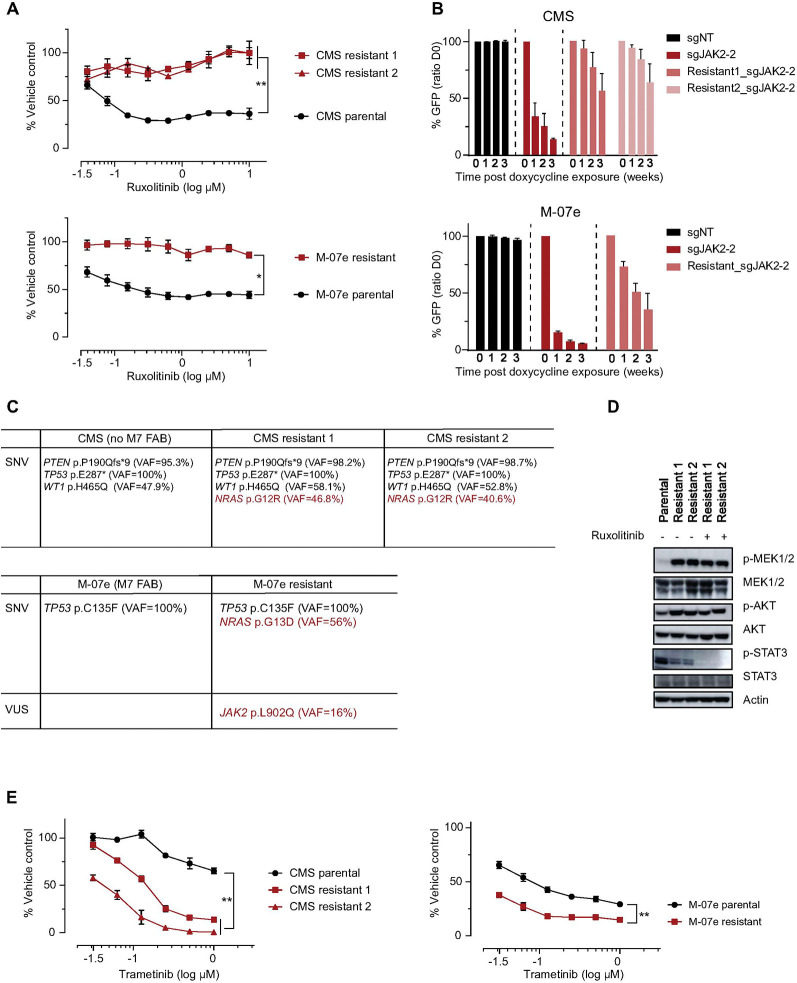
Legend not included with this version

To further study resistance mechanisms to JAK inhibitors in *CBFA2T3::GLIS2* AML, we developed *CBFA2T3::GLIS2* AML cell lines with acquired resistance to JAK inhibitors. We grew CMS and M-07e cells with increasing concentrations of ruxolitinib to a maximum concentration of 1.5 μM (10-fold IC_50_) (Supplemental Fig. 6A). We measured the IC_50_ of ruxolitinib in the resistant cell lines and detected complete resistance in comparison to naive cells (Fig. 6A). These cells were also cross-resistant to tofacitinib (Supplemental Fig. 6B). Generation of these models resistant to JAK enzymatic inhibition also rendered them independent of *JAK2* genetic KO (Supplemental Fig. 6C and Fig. 6B). Indeed, genotyping of these cells revealed that the ruxolitinib-resistant sublines had *NRAS* missense mutations: NRAS p.G12R with a VAF of 46.8 and 40.6% for the CMS sublines and *NRAS* p.G13D with a VAF of 56% for M-07e (Fig. 6C).

We evaluated the main downstream effectors of JAK-STAT and RAS-MAPK-ERK pathways in the resistant cells, and found an increase in phosphorylated MEK and AKT (Fig. 6D, Supplemental Figure S6D), indicating that in the resistant cells, activation of parallel signaling pathways results in resistance to JAK inhibitors. We next exposed these ruxolitinib-resistant cells to the MEK inhibitor trametinib. Although trametinib showed efficacy on the parental cells, the resistant lines were even more sensitive to MEK inhibition consistent with rewiring from a JAK dependency to a RAS-MAPK pathway dependency (Fig. 6E).

### Combined JAK and MEK inhibition improved cytotoxicity in CBFA2T3::GLIS2 AML

We were next interested in whether the combination of JAK and MEK inhibition would have enhanced activity compared to single agent treatments. The combination of trametinib with ruxolitinib in CMS and M-07e parental and resistant cell lines in vitro was synergistic (Supplemental Figures S7 A-B).

**Figure 7:**
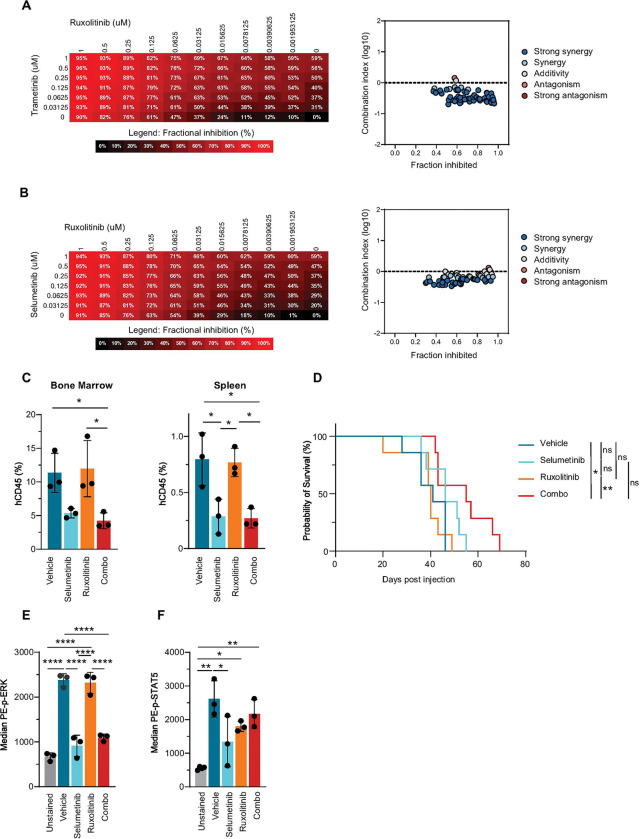
Legend not included with this version

To investigate the effect in vivo, we used the PDX model CPCT-0027, derived from the patient described above, which has a *CBFA2T3::GLIS2* fusion and an NRAS mutation. We first exposed this model to both drugs in vitro. We observed a synergistic effect of ruxolitinib with either trametinib or selumetinib (Fig. 7A and 7B). We next evaluated the impact of selumetinib versus trametinib on ERK phosphorylation as a pharmacodynamic marker in vivo and found that selumetinib was more effective at inhibition of ERK phosphorylation measured by phospho-flow staining (Supplemental Figs. 7C). Selumetinib was thus selected for in vivo testing. Mice were injected with CPCT-0027 cells and engraftment assessed by BMA. Drug treatment started at the earliest evidence of engraftment, 10 days after injection. After 11 days of treatment, the combination of selumetinib and ruxolitinib resulted in a decrease in leukemia cells in the bone marrow and spleen (Fig. 7C). Accordingly, the combination treatment resulted in increased survival in the combo versus vehicle group (41 versus 55 days; p = 0.03) (Fig. 7D). While selumetinib showed a strong impact on pERK as a pharmacodynamic marker in the AML cells (Fig. 7E), mice treated with ruxolitinib, and the combination did not show statistically significant changes by phospho-flow staining for p-STAT5 (Fig. 7F) suggesting that JAK2 signaling was not sufficiently suppressed.

## Discussion

Non-DS-AMKL has a very poor prognosis, particularly in patients with the *CBFA2T3::GLIS2* fusion, which is associated with chemoresistance and aggressive disease. This fusion is known to disrupt key gene expression programs, blocking differentiation and enhancing self-renewal (4, 5). Using genome-scale CRISPR-Cas9 screening, we found this subtype of AML to be dependent on JAK2. Accordingly, JAK2 inhibitors, including ruxolitinib, showed selective efficacy in *CBFA2T3::GLIS2* AML compared to other AML subsets such as *KMT2A*-rearranged. Concordant with our study, Drenberg et al. previously reported a screen of 6568 unique compounds in 8 AML cell lines and revealed selective activity of JAK inhibitors for pediatric AMKL cell lines including those with *CBFA2T3::GLIS2* fusion (44).

Signaling mutations in general play a critical role in the development of childhood leukemia (45). JAK mutations, particularly those affecting *JAK1* and *JAK2*, are oncogenic in both acute lymphoblastic leukemia (ALL) (46,47) and myeloproliferative neoplasms (MPN). Recurrent mutations in the JAK-STAT pathway are also described in *CBFA2T3::GLIS2* fusion AML, observed in 13 to 23% of these patients (4,8, 9). These mutations result in the abnormal activation of the JAK-STAT signaling pathway, which leads to uncontrolled cell proliferation and survival, contributing to leukemogenesis. In ALL, JAK mutations are often associated with high-risk subtypes, such as Ph-like ALL (46). Similarly, in MPNs, *JAK2*V617F is a hallmark mutation that drives the excessive production of blood cells, a key feature of these disorders (48,49). No such outcome association has been established for *CBFA2T3::GLIS2* AML but the rarity of this disease subset makes it challenging to draw definitive conclusions. We provide evidence that in the case of *CBFA2T3::GLIS2* AML, the JAK-STAT pathway is activated even in the absence of mutations in JAKs, particularly in the M7 FAB subset. This activation may explain the sensitivity to JAK inhibitors that we and Drenberg et al. observed (44).

Ruxolitinib has been used in various hematologic conditions, including pediatric leukemias, GVHD, and MPN (50, 51). While ruxolitinib has shown clinical benefit in several settings, there are limitations to its efficacy, with a lack of selectivity, a suboptimal pharmacokinetics profile with a short half-life (52) and the emergence of drug resistance (51). In Drenberg’s publication, ruxolitinib significantly improved survival in a preclinical in vivo setting; however, the disease ultimately progressed, leading to death (44). It is worth noting that the PDX model used by Drenberg et al. harbored a *JAK*V617F mutation, which may explain the greater sensitivity to ruxolitinib observed in their study compared to ours. In our hands, while ruxolitinib demonstrated efficacy in vitro in the PDX cells, it did not have a significant effect on disease burden in vivo, likely due to a poor PK/PD as evidenced by the suboptimal targeting of the STAT pathway. Nonetheless, consistent with our in vitro findings and Drenberg’s work, we observed a clinical response in a patient with *CBFA2T3::GLIS2*, though the disease eventually progressed. The discrepancy between the patient’s clinical response to ruxolitinib and the lack of response observed in the PDX model warrants further discussion. One possibility is that the patient’s response was driven by the combined effect of the azacytidine and ruxolitinib combination, rather than ruxolitinib alone. Another consideration is the potential difficulty in achieving equivalent target engagement in mice, given the pharmacokinetic and pharmacodynamic differences between species. Indeed, targeting the JAK-STAT signaling pathway appeared suboptimal in our PDX study.

Looking to the future, there is optimism for more effective treatment options. Type II JAK inhibitors have advanced into clinical trials and show promise for enhanced selectivity and efficacy over first-generation inhibitors. One such compound, AJ1–11095, is currently undergoing evaluation in a Phase 1 study (NCT06343805) for patients with MPN who failed to response or relapsed after treatment with type I JAK2 inhibitors (54).

Given the complexity of resistance, gaining a deeper insight into the underlying mechanisms is crucial for developing more effective combination therapies. One of the mechanisms of resistance to JAK2 inhibitors is compensatory activation of other signaling pathways. In our study, we found MAPK pathway activation as a mechanism of acquired resistance to ruxolitinib using both CRISPR-Cas9 screening and development of ruxolitinib-resistant sublines. RAS mutations, including those in *NRAS* and *KRAS*, are commonly observed in pediatric AML as well as in ALL, occurring in 6% of AMKL with *CBFA2T3::GLIS2* (8,9). These mutations dysregulate the RAS-MAPK pathway, leading to enhanced cell growth and survival. In certain subsets of AML, RAS mutations can portend a poor prognosis, particularly when they are present alongside other high-risk genetic alterations (55). RAS mutations are notorious for conferring resistance to a variety of targeted therapies, such as SYK and FLT3 inhibitors (56 – 58). The dysregulated RAS-MAPK pathway effectively bypasses the therapeutic blockade of these pathways, allowing leukemic cells to continue proliferating despite treatment. Moreover, RAS mutations can also reduce the effectiveness of epigenetic modulators, including IDH inhibitors, which are designed to target specific epigenetic vulnerabilities in leukemia cells. This common resistance pattern highlights the challenge of treating RAS-mutated leukemias and underscores the need for combination therapies or novel approaches that can overcome these resistance mechanisms.

Emerging treatment aimed at inhibiting aberrant signaling proteins offers hope for improving outcomes for children with leukemia. Recent advancements in RAS-directed therapies and downstream pathway inhibitors, such as MEK inhibitors, may have efficacy in difficult-to-treat pediatric leukemias (59). There is also growing optimism for more potent therapies in the future, aimed at better targeting RAS mutations. Sotorasib and adagrasib, both *KRAS* G12C inhibitors, have demonstrated significant success in clinical trials improving progression-free-survival in non-small cell lung cancer (60,61), while belvarafenib has recently shown promise in targeting *NRAS* mutations in a phase Ib clinical trial including patients with BRAF^V600E^- and NRAS-mutant melanoma (62).

Several studies have explored the combination of JAK and MEK inhibition. In MPN, combining JAK and MEK inhibition has shown a suppression of MEK/ERK activation in *JAK2*V617F and *MPL*W515L mice with increased efficacy and reversal of fibrosis to an extent not seen with JAK inhibitors alone (63). Concordantly, combined inhibition of JAK and MEK effectively controls juvenile myelomonocytic leukemia and a myeloproliferative variant of chronic myelomonocytic leukemia *NRAS*^G12D/G12D^ mice (64). Given the complexity of RAS signaling and the development of resistance to monotherapies, future approaches are likely to involve combination strategies. In *CBFA2T3::GLIS2* AML, combining MEK inhibitors with JAK inhibitors could enhance efficacy and prevent resistance.

FOLR1 targeted therapies are currently being evaluated in the clinic for *CBFA2T3::GLIS2* AML as FOLR1 is expressed on the cell surface in this disease (65). Future combinations might explore targeting the extracellular receptor FOLR1 with inhibition of intracellular signaling pathways such as JAK and MAPK. This strategy might also help address the issue of intratumoral heterogeneity, where subclones of leukemia cells rely on different oncogenic pathways. These combination strategies still need to be explored in preclinical studies to evaluate their efficacy and toxicity profiles.

In summary, while *CBFA2T3::GLIS2* leukemia remains a difficult-to-treat disease, we have validated *JAK2* as a selective dependency offering a new possibility for precision medicine in this very high-risk AML subset. Our work, however, also highlights the difficulty of treating AML with monotherapy, with rapid emergence of resistance. Ongoing research into combinations with JAK inhibitors and other targeted approaches will be critical in developing more effective treatments for this aggressive leukemia.

## Data Availability

The CRISPR-Cas9 drop-out screen data for WSU-AML and the CRISPR-Cas9 drug modifier screen data for CMS and WSU-AML cell lines are publicly available for download at figshare.com upon manuscript publication.
